# LAG3 as a marker of immune activation in esophageal squamous carcinoma treated with concurrent chemoradiotherapy

**DOI:** 10.1007/s00262-025-04076-2

**Published:** 2025-05-24

**Authors:** Yuxuan Zhang, Zijing Zhou, Yuanyuan Rui, Fanhao Kong, Zhoubo Guo, Gang Zhao, Jun Wang, Jiacheng Li, Fangdong Zhao, Hui Huang, Fang Fang, Jiarui Zhang, Tian Zhang, Wencheng Zhang, Ping Wang, Xi Chen, Peng Zhen, Qingsong Pang

**Affiliations:** 1 Department of Radiation Oncology, Tianjin Medical University Cancer Institute & Hospital, National Clinical Research Center for Cancer, Key Laboratory of Cancer Prevention and Therapy, Tianjin’s Clinical Research Center for Cancer, West Huan-Hu Rd, Ti Yuan Bei, Hexi District, Tianjin, 300060 China; 2 Department of Pathology, Tianjin Medical University Cancer Institute & Hospital, National Clinical Research Center for Cancer, Key Laboratory of Cancer Prevention and Therapy, Tianjin’s Clinical Research Center for Cancer, West Huan-Hu Rd, Ti Yuan Bei, Hexi District, Tianjin, 300060 China; 3 Department of Radiotherapy, The Fourth Hospital of Hebei Medical University, Hebei Clinical Research Center for Radiation Oncology, No. 12 Jian Kang Road, Shijiazhuang, 050010 Hebei China; 4 Department of Radiation Oncology, Chifeng Tumor Hospital, Second Affiliated Hospital of Chifeng University, 45 Jiefang Street, Hongshan District, Chifeng, 024000 Inner Mongolia China

**Keywords:** Esophageal squamous carcinoma, Concurrent radiotherapy, Immune checkpoint, Lymphocyte activation gene-3 (LAG3)

## Abstract

**Introduction:**

Esophageal squamous carcinoma (ESCC) is a common malignant tumor of the gastrointestinal tract with high morbidity and mortality rates. Lymphocyte activation gene-3 (LAG3), an important suppressive immune checkpoint in tumor immunity, exhibits a wobbling effect in the prediction of ESCC efficacy.

**Methods:**

Tumor bite paraffin-embedded specimens from 84 patients diagnosed with ESCC, all of whom received radical concurrent chemoradiotherapy (CCRT) at our institution, were screened. For each tissue, we delineated the partitions and analyzed the spatial distribution of the tumor in an in situ immune microenvironment. The density and regional characteristics of immune factor-positive cells, together with the dynamics of various cells based on treatment regimens, were considered important factors influencing the prognostic significance of cancer.

**Results:**

Compared with baseline tissues, the density of CD4 + and CD8 + T cells in the tumor microenvironment of the on-treatment tissues decreased, but the expression of IFN-γ in CD4 + and CD8 + T cells increased. The density of LAG3 positive cells was correlated significantly with the density of CD4 + and CD8 + T cells in both baseline and on-treatment tissues. The density of LAG3 + T cells and the rate of LAG3 positivity in activated CD4 + and CD8 + T cells were associated with elevated Ki67 expression. There was a significant correlation between high LAG3 expression and active CD4 + and CD8 + T cells in tumor cells. Elevated densities and tighter spatial relationships of both CD4 + and CD8 + T cells were associated with longer overall survival with ESCC.

**Conclusion:**

Concurrent chemoradiotherapy without combined immunotherapy inhibited tumor-infiltrating T cells to a certain extent, and elevated immune checkpoint LAG3 was closely associated with immune activation in the ESCC tumor microenvironment.

**Supplementary Information:**

The online version contains supplementary material available at 10.1007/s00262-025-04076-2.

## Introduction

Esophageal cancer is a common gastrointestinal malignant tumor. The number of new cases of esophageal tumors in 2016 in China was approximately 252.5 thousand and there were 193.9 thousand deaths, which is the fifth highest globally [1]. Due to the enormous patient base of esophageal tumors in China, simple surgical regimens or neoadjuvant radiotherapy, as well as radical radiotherapy for inoperable patients, continue to be the mainstay treatment [2]. Although new cases and deaths from esophageal cancer have declined significantly over the past few years with the introduction of more standardized diagnostic and treatment protocols, the numbers are still very high [2].

Lymphocyte activation gene-3 (LAG3), an important inhibitory immune checkpoint in tumor immunity, is expressed on activated CD4 + and CD8 + effector and exhausted T cells, natural killer (NK) cells, and B cells induced by T cells [3, 4]. Aberrant expression of LAG3, which is interdependent with its ligand FGL-1 and inhibits T-cell function, has been reported to be negatively associated with clinical prognosis in a variety of cancers, including solid tumors and some cancers of hematological origin. This effect is influenced by PD-1, which is also an inhibitory immune checkpoint [5, 6].

Combined radiotherapy has been found to induce immunogenic cell death and enhance the efficacy of immunotherapy [6, 7], and immune checkpoint blockade immunotherapy administered concurrently with chemotherapy and/or radiotherapy has been suggested to provide additional benefits for Esophageal squamous carcinoma (ESCC) or other epithelial tumors [8–10]. Clinical trials combining two immune checkpoints (LAG3 and PD-1) for previously untreated metastatic or unresectable melanomas have demonstrated the feasibility of anti-LAG3 treatment [11].

Evidence suggests that the number of tumor-infiltrating lymphocytes (TIL), particularly activated CD4 + and CD8 + effector T cells, improves the prognosis of ESCC patients [12]. Several ESCC tumor microenvironment studies, predominantly targeting the immune checkpoint PD-1, have demonstrated the predictive potential of the content and spatial interrelationships of active immune cells in TIL for the treatment of malignancies [10, 13, 14]. Although similar studies have investigated LAG3's function, they have failed to reach conclusive findings. Recent survival analyses have demonstrated tumor-specific effects of LAG3 expression on overall survival across various malignancies, including esophageal carcinoma, breast cancer, and malignant melanoma [15–18]. Notably, even within ESCC (esophageal squamous cell carcinoma), conflicting studies have reported both favorable and unfavorable prognostic associations with LAG3 expression [15, 16]. Compared to the PD-1 immune checkpoint, which has gained popularity in recent years, LAG3 has a less defined biological role, and a consensus on how it mediates T-cell inhibitory signaling in different cells has yet to be proposed [19]. Furthermore, it has been predicted that LAG3 expression can directly affect PD-1 responsiveness to therapy and patient prognosis [20]. In our analysis, we incorporated comprehensive evaluation of immune cell composition and spatial distribution within the tumor microenvironment (TME) both pre- and during treatment. Given LAG3's unique characteristics compared to other co-inhibitory receptors, we specifically examined its potential as a predictive biomarker and local immunomodulatory factor in TME, with the goal of refining therapeutic strategies and improving outcomes for locally advanced ESCC patients. Therefore, in the present study, we investigated ESCC patients with different activation profiles of LAG3 and TIL and analyzed their prognostic value in tumor therapy.

## Materials and methods

Patients (N = 84) diagnosed with esophageal squamous cell carcinoma (ESCC) and treated with radical concurrent radiochemotherapy at Tianjin Medical University Cancer Institute & Hospital from June, 2015 to March, 2019 were taken into the study. The follow-up duration of the survivors ranged from 98 to 2323 days (median 1232.5 days). We enrolled patients with ESCC originally scheduled for primary treatment according to neoadjuvant synchronous radiotherapy, who underwent ultrasonographic endoscopy and biopsy when the total dose of radiation therapy reached 40 Gy, and were evaluated to be inoperable and then converted to radical concurrent radiochemotherapy. According to the guideline, the final total radiotherapy dose was 50.4 Gy/28 f, and the chemotherapy was mainly paclitaxel or fluorouracil in combination with cisplatin regimens, both of which were administered for 5 cycles. Clinical stages were defined according to the 8 th edition of the American Joint Committee on Cancer (AJCC)-TNM Classification. The clinical features of ESCC patients are shown in Table 1. All patients analyzed received no other anti-tumor treatments before, during, or after CCRT. All cohort patients either achieved 5-year survival or ultimately died from ESCC (esophageal squamous cell carcinoma) or its complications. The cohort excluded patients who: Did not complete the CCRT regimen; Received additional anti-tumor therapies; Died from accidental causes; Were lost to follow-up.

**Table 1:** Baseline status of patients included in the study

	mIHC (n = 84)	scRNA-seq (n = 10)
Age		
Median, IQR	64 (59–67)	67.5 (58.75–69.25)
Age Group		
≥ 65	37 (44.0%)	6 (60.0%)
< 65	47 (56.0%)	4 (40.0%)
Gender		
Male	71 (84.5%)	7 (70.0%)
Female	13 (15.5%)	3 (30.0%)
Smoking		
Ever-smoking	62 (73.8%)	9 (90.0%)
Never-smoking	20 (23.8%)	1 (10.0%)
Non-recorded	2 (2.4%)	0 (0.0%)
Drinking		
Ever-drinking	61 (72.6%)	7 (70.0%)
Never-drinking	20 (23.8%)	3 (30.0%)
Non-recorded	3 (3.6%)	0 (0.0%)
Location		
Cervical	3 (3.6%)	0 (0.0%)
Upper Thor	12 (14.3%)	0 (0.0%)
Middle Thor	39 (46.4%)	2 (20.0%)
Lower Thor	29 (34.5%)	8 (80.0%)
EGJ	1 (1.2%)	0 (0.0%)
Tumor length		
Median, IQR	5.00 (3.75,7.00)	Non-recorded
T		
1	8 (9.5%)	0 (0.0%)
2	1 (1.2%)	0 (0.0%)
3	52 (61.9%)	10 (100.0%)
4	23 (27.4%)	0 (0.0%)
N		
0	18 (21.4%)	5 (50.0%)
1	45 (53.6%)	3 (30.0%)
2	21 (25.0%)	2 (20.0%)
M		
0	83 (98.8%)	10 (100.0%)
1	1 (1.2%)	0 (0.0%)
TNM stage		
I	8 (9.5%)	0 (0.0%)
II	10 (11.9%)	5 (50.0%)
III	43 (51.2%)	5 (50.0%)
IV	23 (27.4%)	0 (0.0%)

*IQR* interquartile range, *EGJ* gastroesophageal junction

The tissue specimens of each patient before and during (when radiotherapy doses were up to 40 Gy) therapy were obtained using biopsy punch forceps in endoscopic examinations and the formalin-fixed, paraffin-embedded method. Tyramide signal amplification (TSA)-based multiplex immunohistochemistry (mIHC) staining was used to monitor the distribution of cancer and immune cells in the TME. Table 2 shows the antibodies used for staining and manufacturer’s instructions. For all tissue sections, consecutive slices were subjected to both hematoxylin–eosin (HE) staining and multiplex immunohistochemistry (mIHC). Following staining: Two qualified pathologists independently reviewed HE-stained sections. Normal epithelial regions and necrotic tissue were digitally annotated and excluded using specialized software.Tumor-stroma boundaries were clearly demarcated. The annotated regions from HE-stained sections were then aligned and integrated with corresponding mIHC-stained sections for subsequent analysis.

**Table 2:** Antibodies and staining solutions

Antibody	Catalog number	Brand	Dilution ratio	TSA reagent
PAN-CK	GM351507	Gene Tech	1:2	Opal 520
CD4	ab133616 [41]	Abcam	1:1000	Opal 690
CD8	ab17147 [42]	Abcam	1:50	Opal 620
LAG3	ab180187 [43]	Abcam	1:500	Opal 570
Ki-67	9449 T [44]	Cell Signaling Technology	1:1000	Opal 570
IFN-gamma	MAB2853 [45]	R&D Systems	1:100	Opal 650

### Image and statistical analysis

**Fig. 1:**
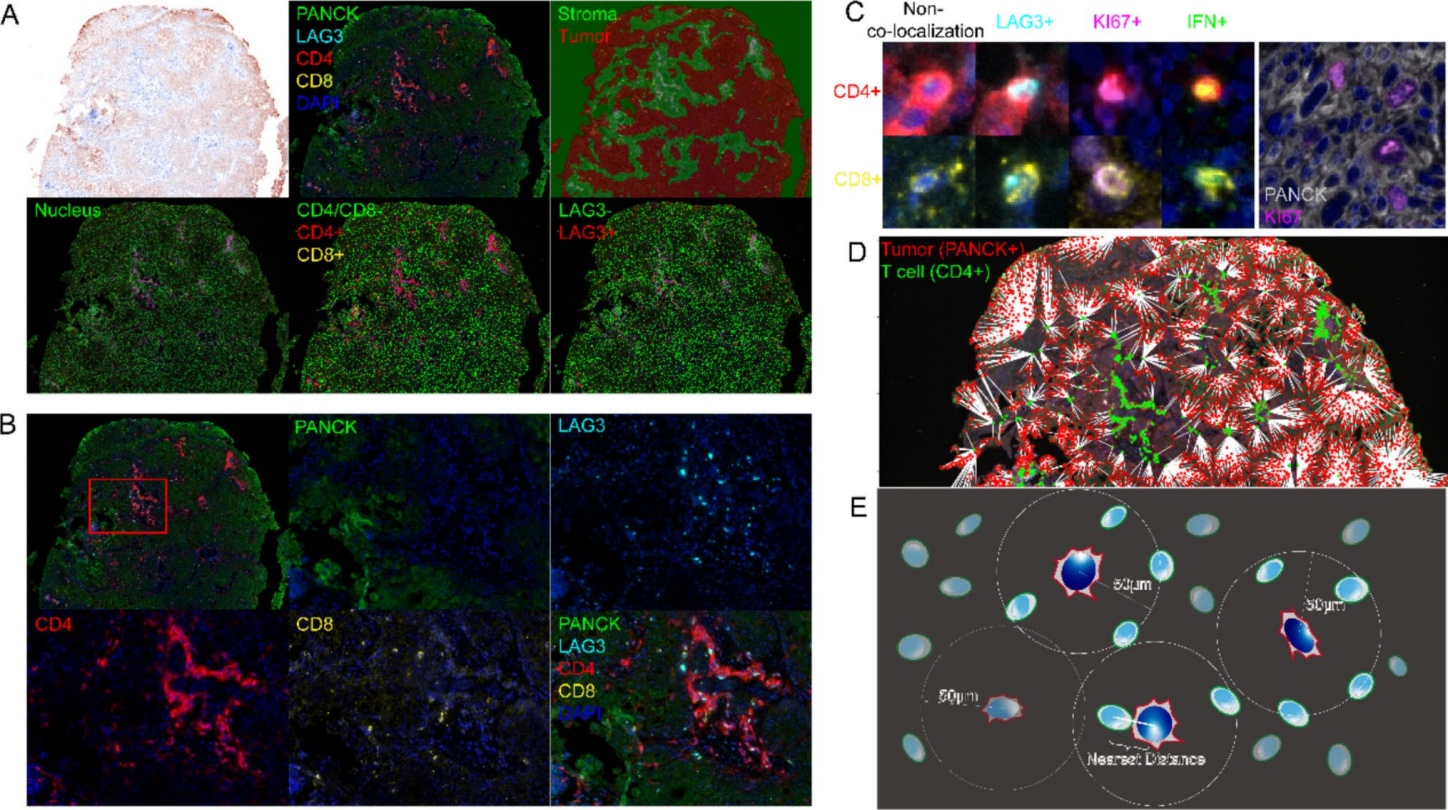
Identification and characterization of tumor-infiltrating immune cells in esophageal cancer tissues. **A** Image analysis pipeline including pseudo brightfield, tumor and stromal region segmentation, nuclei localization and positive/negative expression views, CD4/CD8 and LAG3 as illustrations. **B** Schematic representation of different channels of a multicolor fluorescence section, including merge view and single channel. The upper left corner shows the full slice view, and the other five images show a schematic of individual versus merged markers in the red square portion. **C** Examples of co-localization situations on the slice that need to be included in this study. **D** Schematic diagram of spatial location relationships, using CD4 + as an example. **E** Schematic diagram of parameters required for spatial analysis

Tumor (T) and stromal (S) regions were segmented using software. For further analysis, cells with different immunophenotypes are processed, exhibit software-recognizable fluorescence on sections, and are visualized and datamined by software guided by predefined protocols (Fig. 1A). The cells stained with DAPI were manually classified based on images containing a single fluorescence signal in the spectral library (Fig. 1B). Because of the properties of mIHC staining, we were able to localize cells with co-expression properties in this step (Fig. 1C). The central cells were paired with the surrounding cells in radii of 50 μm, and a network including the central tumor cells and the surrounding immune cells was established (Fig. 1D). We obtained the minimum and median distances from the surrounding immune cells to the central tumor cells, and the average number of specific immune cells within the specified distance of each tumor cell (Fig. 1E). During preliminary experiments, we performed conventional immunohistochemical (IHC) staining more than three times on consecutive sections using antibodies from different manufacturers. Compared results between conventional IHC and multiplex IHC (mIHC) to optimize the final mIHC protocol. For each marker, selected six pre-stained positive and six negative specimens. Determined intensity cutoff values using INFORM® and HALO® image analysis software. For all the factors analyzed, between-group comparisons were performed using Wilcoxon signed-rank tests. Correlations were assessed using Spearman's rank correlation analysis. Optimal cutoff values for each factor were determined through ROC curve analysis to stratify high/low expression groups in K-M survival analysis. All statistical analyses were performed using R (version 4.3.0) and IBM SPSS Statistics (version 26).

Single-cell RNA sequencing data processing and analysis were performed using Cell Ranger (v7.2.0, 10 × Genomics) to align raw sequencing reads to the human reference genome and generate gene expression matrices for each sample. The filtered feature-barcode matrices were subsequently integrated and analyzed using the Seurat package (v5.1.0) implemented in R (v4.3.0). Quality control filtering was applied to remove low-quality cells based on the following criteria: cells expressing fewer than 150 genes and cells with mitochondrial UMI content exceeding 15%. Then the data were normalized using the NormalizeData function with log-normalization, followed by identification of highly variable genes through FindVariableFeatures. Data scaling was performed using ScaleData to account for technical variations. Principal Component Analysis (PCA) was conducted on the scaled data, with the top 15 dimensions selected for downstream analysis based on elbow plot evaluation. To address batch effects across samples, Harmony was employed to correct the PCA-reduced dimensions. The batch-corrected dimensions were then utilized for non-linear dimensionality reduction (t-SNE) and subsequent cell clustering. Finally, cell populations were visualized using t-SNE projections and annotated based on marker gene expression patterns.

## Results

### T-Cell Infiltration Discrepancy before and during treatment

**Fig. 2:**
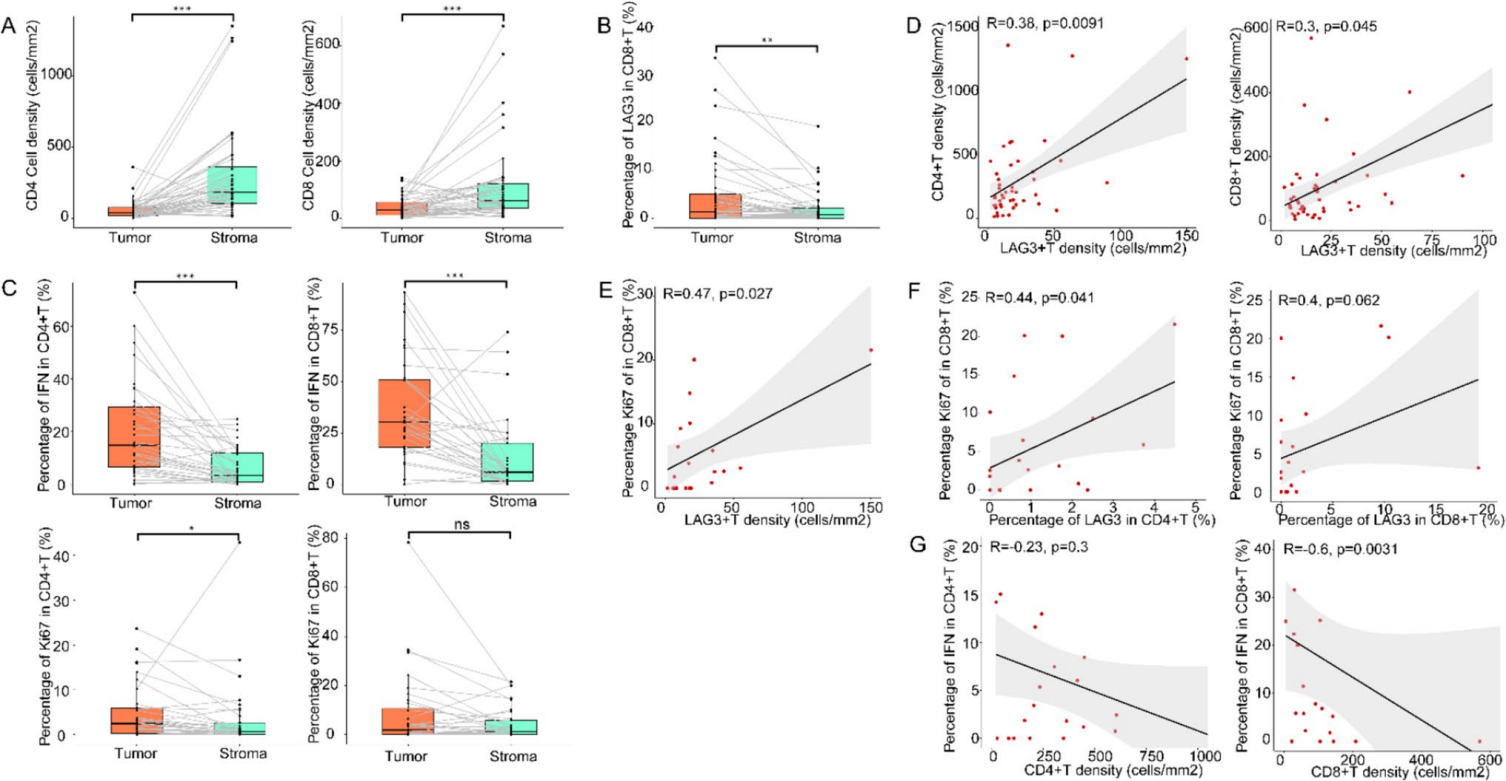
Composition of T cell subpopulations distributed in tissues. **A** Densities of CD4 + T cells and CD8 + T cells, **B** percentage of LAG3 + in CD8 + T cells, **C** percentage of IFN + and Ki67 + in CD4 + and CD8 + T cells within tumor regions and stromal regions at baseline. **D** Relationship between CD4 + T and CD8 + T cell density and LAG3 + T cell density in stromal tissues at baseline. Relationship between Ki67 positivity of CD8 + T cells in stromal tissues at baseline and **E** LAG3 + T cell density, **F** LAG3 positivity in CD4 + and CD8 + T cells. **G** Relationship between IFN positivity and cell density in stromal CD4 + and CD8 + cells at baseline. Wilcoxon signed-rank tests in (**A**-**C**), Spearman tests in (**D**-**G**), p < 0.05, statistically significant

In the baseline group, we first delineated and compared the tumor and stromal parts of the cancerous tissues. Compared with the tumor regions, higher CD4 + and CD8 + T cell densities were observed in the stromal area (183.95/mm2 vs. 40.03/mm2, p < 0.001; CD4 + : 61.80/mm2 vs. 29.57/mm2, p < 0.001; CD8 +, Fig. 2A). CD8 + T cell population in the stroma had a lower LAG3 ratio (0.71% vs. 1.29%, p = 0.003; Fig. 2B), whereas CD4 + T cells did not show this property. Although the density and percentage of LAG3 + T cells were uniform throughout the tissues, both CD4 and CD8 positive T cells in the tumor portion have higher Ki67 and IFN-γ expression rates(2.46% vs 0.71%, p = 0.017, Ki67 in CD4 + T; 1.89% vs 1.20%, p = 0.067, Ki67 in CD8 + T; 14.91% vs 3.41%, p < 0.001, IFN in CD4 + T; 30.22% vs 5.88%, p < 0.001, IFN in CD8 + T, Fig. 2C), even though these cells occupied a lower density compared to the stromal part.

We found that the density of LAG3 + T cells was correlated with CD4 + or CD8 + T cells in the local tumor tissues of untreated patients, the correlation was mainly in the stromal zones (Spearman ρ = 0.384, p = 0.009, CD4 +; Spearman ρ = 0.300, p = 0.045, CD8 +, Fig. 2D), rather than among the infiltrating T cells in the tumor area, the core of the cancer nests consisting of PANCK-staining cells. The Ki67 ratio in CD8 + T cells are positively associated with not only the density of LAG3 positive cells in tissues (Spearman ρ = 0.472, p = 0.027, Fig. 2E) but also with the positive rate of LAG3 in both CD4 + and CD8 + T cells (Spearman ρ = 0.440, p = 0.041, CD4; Spearman ρ = 0.404, p = 0.062, CD8, Fig. 2F). However, no significant correlation was detected between the Ki67 + ratio of CD4 + T cells and the positive expression rate of LAG3 in T cells in CD4 + and CD8 + subgroups. In addition, the density of CD8 + T cells in the stromal zones was determined to have an inverse correlation with the percentage of IFN-γ(Spearman ρ = −0.601, p = 0.003), whereas the CD4 + T group similarly did not show this correlation (Fig. 2G). When we focused on the total of baseline tissues, similar to the stromal area, we observed a correlation between densities of LAG3 + T and CD4 + T cells (Spearman ρ = 0.247, p = 0.047, Fig. S1A). The level of LAG3 + T cells in tissues also showed a positive correlation with IFN + CD8 + T cell content (Spearman ρ = 0.404, p = 0.045, Fig. S1B). The positive rate of LAG3 in CD8 + T subpopulation was correlated with the density of CD8 + T cells expressing IFN or Ki67(Spearman ρ = 0.377, p = 0.063, IFN + CD8 + T; Spearman ρ = 0.458, p = 0.024, Ki67 + CD8 + T, Fig. S1C). However, the CD4 + T cells did not show the above-mentioned characteristics.

**Fig. 3:**
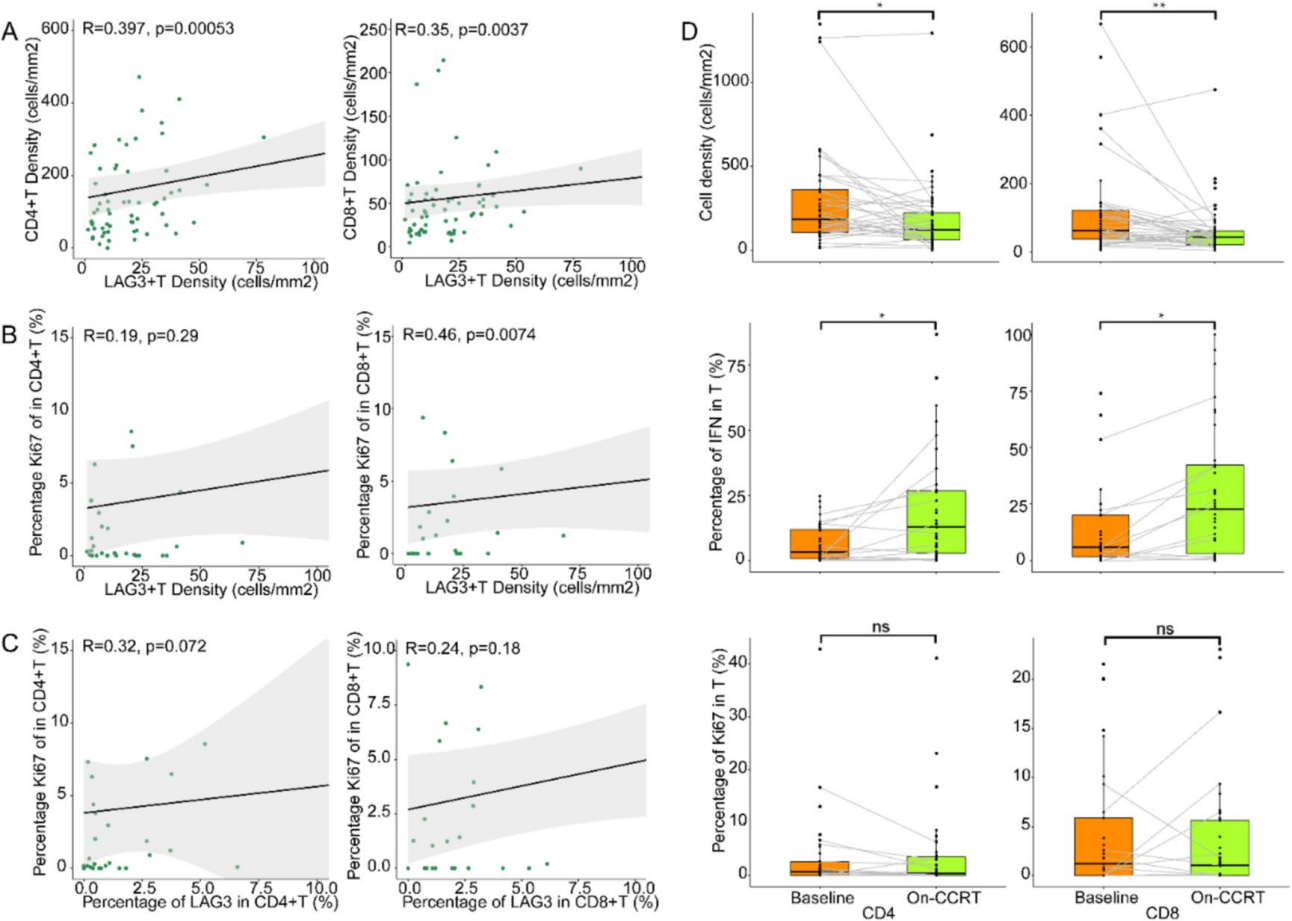
Analysis of T-cell distribution in tissues on-treatment. Relationship between **A** densities, **B** positivity of Ki67 of CD4 + and CD8 + T cells and LAG3 + T-cell densities on-treatment. **C** Relationship between Ki67 positivity and LAG3 positivity in CD4 + and CD8 + T cells on-treatment. D Cells densities, IFN-γ expression rates, and Ki67 expression rates of CD4 + and CD8 + T cells at baseline vs. on-treatment. Wilcoxon signed-rank tests in (**A**-**C**), Spearman tests in (**D**), p < 0.05, statistically significant

Next, we examined on-treatment tissues. As the patients had been treated with a 40 Gy dose of concurrent chemoradiotherapy (CCRT) and there was disruption in the tissue structure, we artificially excluded necrotic areas in the tissue, and not all specimens retained the appropriate tumor area. We found that density of LAG3 + T cells showed a higher correlation with both CD4 + and CD8 + T cells (Spearman ρ = 0.397, p = 0.001, CD4 +; Spearman ρ = 0.351, p = 0.004, CD8 +, Fig. 3A). The content of LAG3 + T cells showed a correlation with the expression rate of Ki67 in CD8 + T cells, but this correlation was not present in the CD4 + T population (Spearman ρ = 0.498, p = 0.004, Fig. 3B). Interestingly, there was no correlation between the rate of LAG3 and Ki67 expression in CD8 + T cells population, but the positive percentage of Ki67 in CD4 + T cells was related to LAG3 positive ratio in on-treatment specimens (Spearman ρ = 0.317, p = 0.072, Fig. 3C). This situation was in contrast to the tendency in the baseline tissues, where the CD8 + T cell subpopulation, but not the CD4 + T cells, exhibited this consistency.

Concurrent chemoradiotherapy affects the immune microenvironment of tumors and surrounding tissues, as reported in many studies. We noticed that in the stromal zones, a decrease was confirmed in terms of the densities of CD4 and CD8 positive T cells after CCRT treatment (122.59/mm^2^ vs. 183.95/mm^2^, p = 0.028, CD4 +; 42.54/mm^2^ vs. 61.80/mm^2^, p = 0.009, CD8 +). We then turned our attention to IFN-γ, whose density (99.67/mm^2^ vs. 23.50/mm^2^, p = 0.004) and expression rate in CD4 + and CD8 + T cells were significantly increased during therapy (12.86% vs. 3.41%, p = 0.035, CD4 + T; 22.89% vs. 5.88%, p = 0.013, CD8 + T). Ki67 was measured to assess T-cell proliferation. However, Ki67 expression in the two TIL subpopulations did not show significant changes (Fig. 3D). Unfortunately, the trend in the proportion of CD4 + and CD8 + T cells among LAG3 cells was not conclusive.

### Regional immune cell infiltration associated with overall survival

**Fig. 4:**
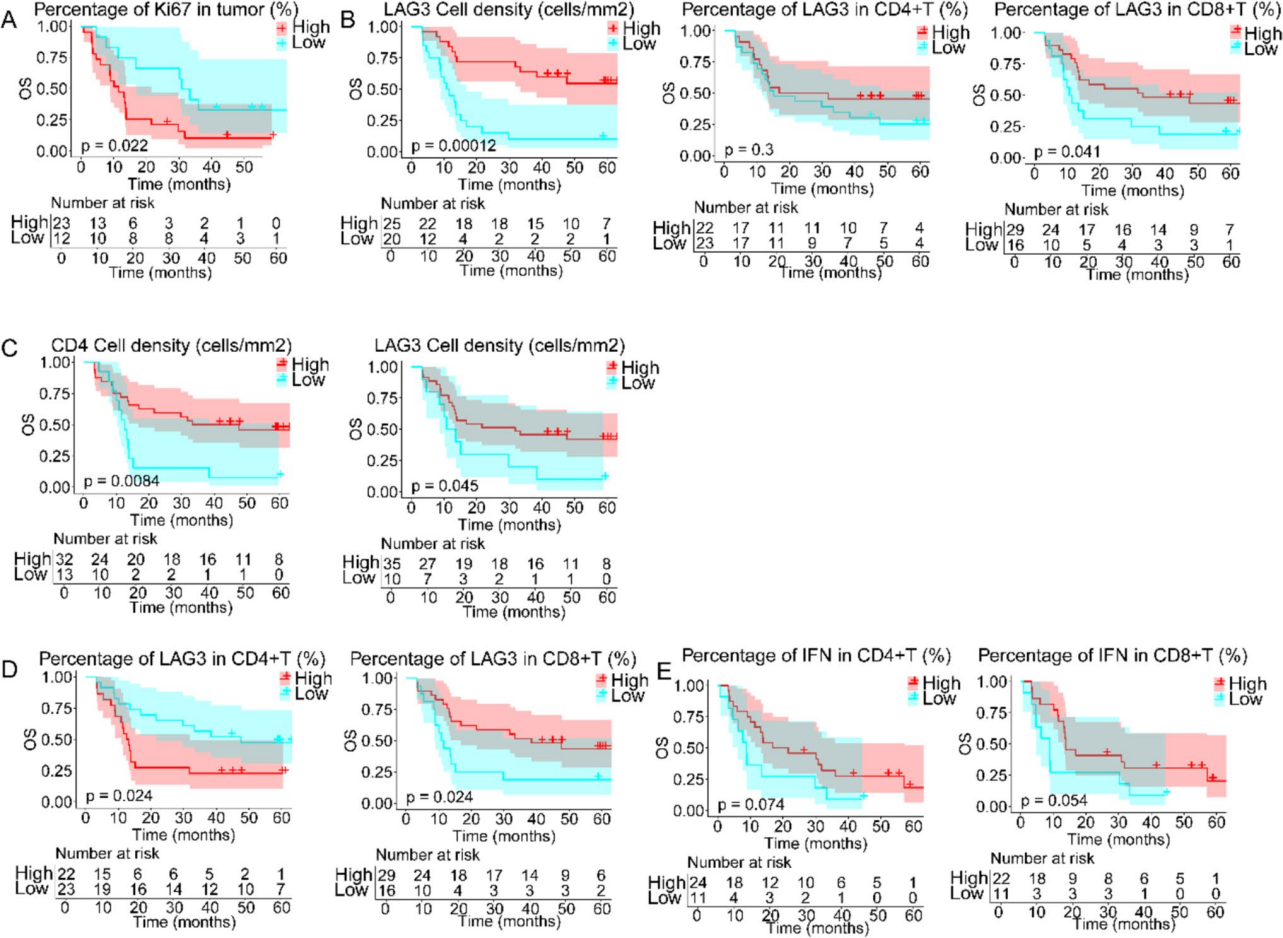
Kaplan–Meier curves showing overall survival based on the distribution of T cells in intratumoral tissues at baseline. Overall survival of patients based on **A** Ki67 positivity of tumor cells, **B** density of LAG3 + T cells and LAG3 expression of CD8 + T cells in tumor regions. Overall survival of patients based on **C** density of CD4 + T and LAG3 + T cells and percentage of CD4 + and CD8 + T cells expressing **D** LAG3 and **E** IFN-γ in stromal regions. The individual TILs were divided into high (redline) or low content (cyanline). Log-rank test was used. A two-sided P < 0.05 was considered statistically significant

In this study, we combined multiplex immunohistochemistry (mIHC) data with continuous follow-up of clinical patients to explore the relationship between the expression of immune-related cytokines in the tumor microenvironment and the prognosis of concurrent chemoradiotherapy. It was intuitive that when Ki67 expression in tumor cells increased in pre-CCRT tissues, as indicated by PANCK staining, the overall survival time was reduced after the initial diagnosis (33.3 vs 11.6 months, p = 0.022; Fig. 4A). The number of T cells within the tumor area had little effect on the overall survival time, and not CD4 + or CD8 + cells, but the density of LAG3 + T cells showed a strong correlation with prognosis (43.0 vs 9.1 months, p < 0.001). Higher LAG3 positive percentage was also beneficial for patients’ overall survival, and this effect was strong in CD8 + T cells (NR vs 13.9 months, p = 0.041) but not in CD4 + T cells (Fig. 4B). A satisfactory positive correlation between overall survival and the density of CD4 positive TILs (40.5 vs 12.5 months, p = 0.008) in the stroma surrounding untreated tumors was observed, as well as LAG3-expressing CD4 + T cells (31.8 vs 12.2 months, p = 0.045, Fig. 4C). Next, the divergent effects were driven by different T-cell subpopulations; higher LAG3 content in CD4 + T cells was related to shorter survival (12.8 vs 47.6 months, p = 0.024), but the expression of LAG3 among CD8 + T cells showed the opposite effect (38.4 vs 11.2 months, p = 0.024; Fig. 4D). The function brought about by the percentages of IFN-γ in CD4 + and CD8 + T cells had the same trend, and those increased proportion could predicted benefit to patients (19.22 vs 8.83 months, p = 0.074, CD4 +, 13.8 vs 8.8 months, p = 0.054, CD8 +, Fig. 4E).

**Fig. 5:**
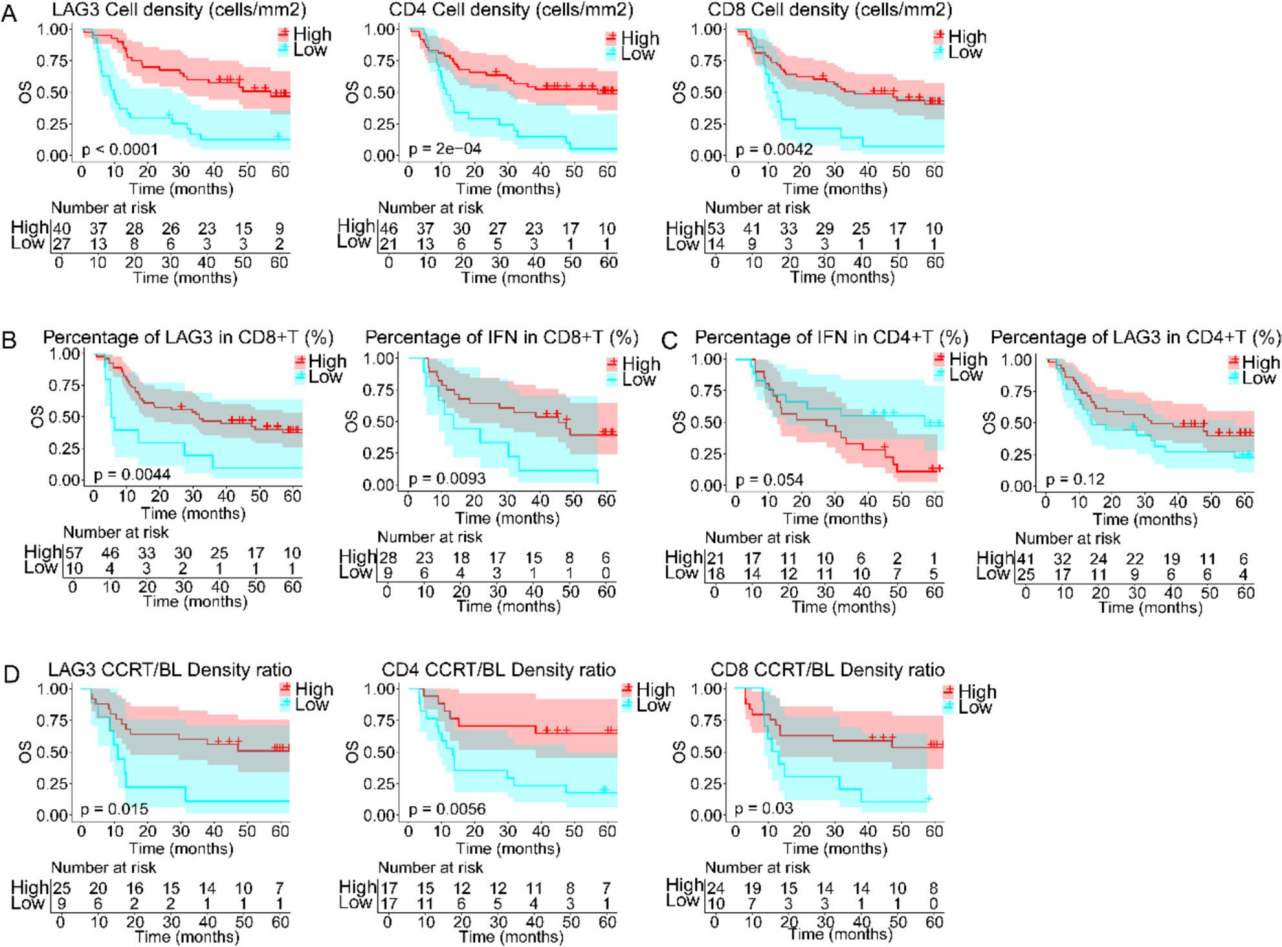
Kaplan–Meier curves showing overall survival based on the distribution of T cells in intratumoral tissues on-CCRT. Overall survival of patients based on **A** densities of LAG3 + T, CD4 + T and CD8 + T cells, **B** LAG3 and IFN-γ expression of CD8 + T cells and **C** IFN-γ expression of CD4 + T cells. **D** Overall survival based on CCRT/BL density ratios of LAG3 +, CD4 +, and CD8 + T cells. The individual TILs were divided into high (redline) or low content or ratio (cyanline). Log-rank test was used. A two-sided P < 0.05 was considered statistically significant

The analysis of on-treatment tissue sections showed that the density of LAG3 + T and CD4 + or CD8 + T cells in patients after CCRT was highly consistent, and all of them showed positive correlation properties on prognosis (57.1 vs 11.4 months, p < 0.001, CD4; 36.0 vs 12.0 months, p = 0.004, CD8; 57.1 vs 9.8 months, p < 0.001, LAG3, Fig. 5A). The expression ratio of both LAG3 and IFN-γ within CD8 + T cells still showed a positive correlation with survival (31.8 vs 5.9 months, p = 0.004, LAG3; 45.2 vs 13.5 months, p = 0.009, IFN, Fig. 5B). In CD4 + TIL, despite the percentage of LAG3 had less effect, the increasement of IFN-γ content was perceived to led to shorter overall survival (27.4 vs 57.1 months, p = 0.054, Fig. 5C).

Baseline tissue was paired with on-treatment tissue from the same patient, the variation ratios of the densities of CD4 +, CD8 +, and LAG3 + T cells in the stromal regions were calculated, and the relationship between these values and survival was determined. The results showed that higher rates of CCRT/BL density ratios of CD4 +, CD8 +, and LAG3 + T cells represented a good prognosis (NR vs 13.4 months, p = 0.006, CD4; NR vs 12.4 months, p = 0.030, CD8; 38.4 vs 11.2 months, p < 0.001, LAG3, Fig. 5D).

### Peritumoral LAG3 spatial distribution predicts immune activation and better outcome

**Fig. 6:**
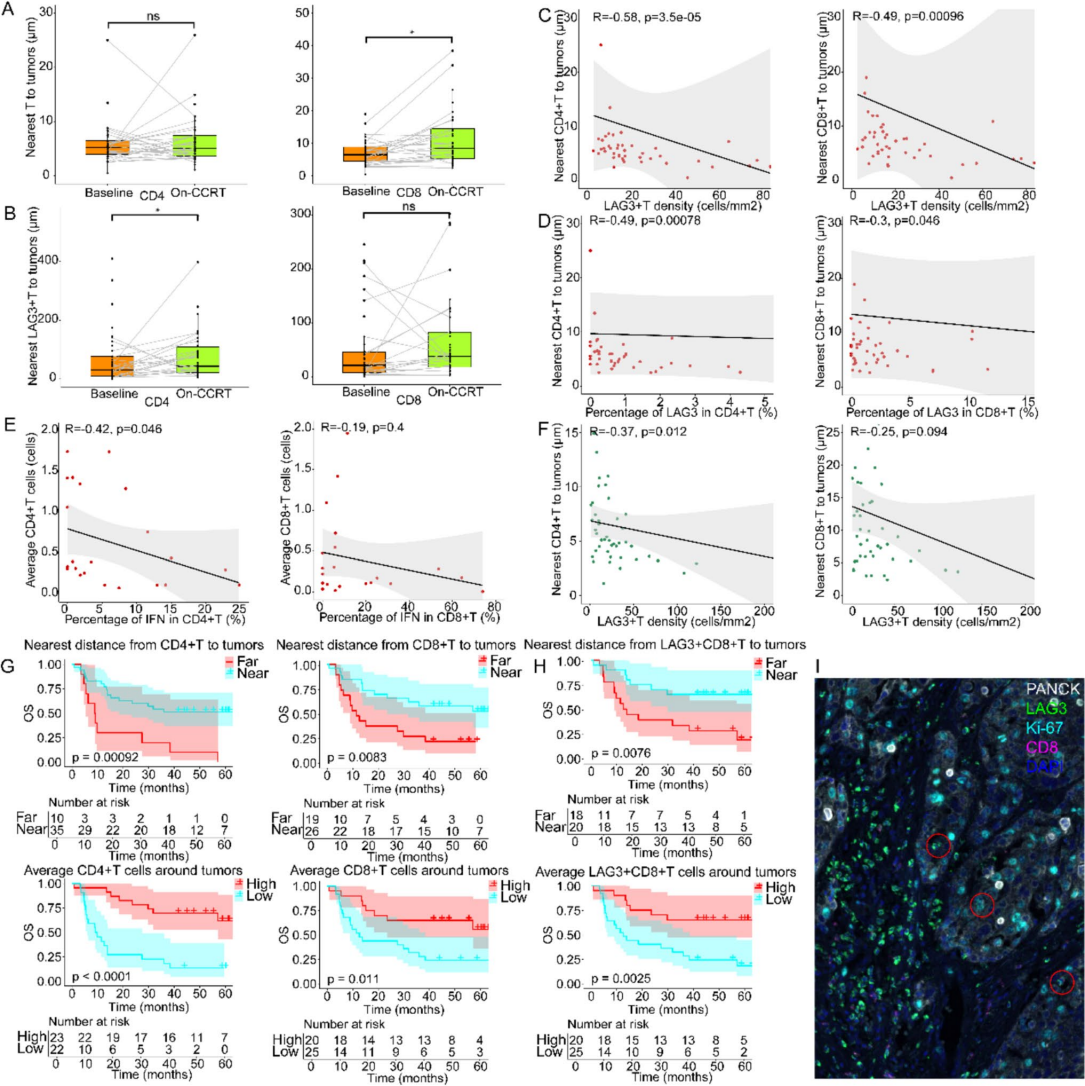
Spatial distribution of T cells in tissues within the tumor microenvironment. **A** Nearest distances from CD4 + and CD8 + T cells to tumor cells and **B** nearest distances from LAG3 + CD4 + and LAG3 + CD8 + T cells to tumor cells at baseline vs. on-treatment. Relationship between nearest distances from CD4 + and CD8 + T cells to tumor cells and **C** density of LAG3 + T cells and **D** the percentage of LAG3 in CD4 + and CD8 + T cells at baseline. **E** Relationship between the average number of T cells around tumor cells at baseline and their IFN-γ expression rate. **F** Relationship between nearest distances from CD4 + and CD8 + T cells to tumor cells and density of LAG3 + T cells on-treatment. Overall survival of patients based on **G** nearest distances from CD4 + and CD8 + T cells to tumor cells and the average number of T cells around tumor cells on-CCRT, **H** nearest distances from LAG3 + CD8 + T cells to tumor cells and the average number of LAG3 + CD8 + T cells around tumor cells on-CCRT. **I** Schematic containing tumor boundaries with T-cell populations, with red circles illustrating infiltrating LAG3 + T cells in the region of the cancer nests. Wilcoxon signed-rank tests in (**A**-**B**). Spearman tests in (**C**-**F**). The individual TILs were divided into high content or far distances (redline) or low content or near distances (cyanline). Log-rank tests in (**G**-**H**). p < 0.05, statistically significant

To further explore the interaction between tumor cells and immune cells, we quantified their spatial relationship in tissue sections, including the distance from each tumor cell to the nearest CD4 + or CD8 + T cell and the average number of immune cells within 50 μm of each tumor cell, and performed overall survival analysis. As CCRT proceeded, the minimum distance between the tumor and peripheral immune cells increased, accompanied by a decrease in the average number of immune cells around the tumor. The trend among CD8 + T subpopulation was relatively prominent (6.46 μm vs 8.49 μm, p = 0.003, Nearest distance; 0.184 vs 0.115, p = 0.054, Average cells), instead of which in CD4 + TILs (Fig. 6A, S2 A). We also discovered that LAG3 + T cells stayed away from tumor cells after treatment, particularly LAG3 + CD4 + T cells, whose changes of minimum distance to tumor cells were statistically significant (31.01 μm vs 42.98 μm, p = 0.023, Fig. 6B).

We assessed the density of LAG3 positive T cells within each baseline tissue and performed paired comparisons with the corresponding CD4 + T cell spatial locations. The results suggested that the density of LAG3 positive T cells and the closest distance of T cells to the tumor showed a significant negative correlation (Spearman ρ = −0.582, p < 0.001, CD4; Spearman ρ = −0.485, p < 0.001, CD8, Fig. 6C), and similarly, there was also a positive correlation with the average number of T cells surrounding the tumor cells (Spearman ρ = 0.446, p = 0.002, CD4; Spearman ρ = 0.311, p = 0.040, CD8, Fig. S2B). We also found that the percentage of LAG3 in CD4 + and CD8 + T cells negatively related to the nearest distance between tumor and corresponding subsets T cells (Spearman ρ = −0.488, p < 0.001, CD4; Spearman ρ = −0.302, p = 0.046, CD8, Fig. 6D), but there was no evidence to show the relationship between LAG3 positive ratio and the average number of peritumor T cells. There was a negative correlation between the ratio of IFN-γ in CD4 + T cells in baseline tissues and the average CD4 + T cells number around the tumor (Spearman ρ = −0.421, p = 0.046), but no trend was observed within CD8 + T cell subsets (Fig. 6E).

In subsequent studies of on-treatment tissues, we found results largely consistent with those obtained in baseline, namely that LAG3 + T cells density negatively correlated with the nearest distance to tumor and positively related to average number of T cells around tumor cells (Nearest distance: Spearman ρ = −0.372, p = 0.012, CD4; Spearman ρ = −0.253, p = 0.094, CD8, Fig. 6F; Average cells: Spearman ρ = 0.511, p < 0.001, CD4; Spearman ρ = 0.409, p = 0.005, CD8, Fig. S2 C). The percentage of LAG3 in each T cell subset no longer showed an obvious correlation with any spatial relationship among the tumor microenvironments, nor was there a correlation between IFN-and the average number of peritumoral T cells found in on-CCRT tissues.

Next, we analyzed the effect of the distribution of immune cells in the tumor microenvironment on patient prognosis. Regarding the spatial relationship of local immune cells in the tumor tissue of the baseline patients, the following trends were observed: the group of patients with a closer distance between T cells and tumor cells had relatively longer overall survival after CCRT administration, but when the immune checkpoint LAG3 was included in the study, the farther distance from tumors to the nearest LAG3 positive CD4 + or CD8 + T cells resulted in better but statistically insignificant prognosis (Fig. S2D, E). When we focused on the CCRT tissues, this trend changed. T cells closer to the tumors (NR vs 9 months, p < 0.001, CD4; NR vs 11.4 months, p < 0.001, CD8) and more TILs around the tumor cells (NR vs 9.4 months, p < 0.001, CD4; NR vs 12.6 months, p = 0.011, CD8, Fig. 6G) prolonged patient overall survival, even if LAG3 expression was considered, and CD8 + T cells possessed a proximate effect as well (NR vs 13.2 months, p = 0.007, Nearest distance; NR vs 12.6 months, p = 0.003, Average cells, Fig. 6H). However, the spatial relationship between CD4 + T cells and tumors did not significantly alter prognosis (Fig. S2 F). Given the association between high LAG3 expression and favorable prognosis, these T cells likely possess enhanced anti-tumor capabilities. Our analysis revealed that CD4 + and CD8 + T cells in closer proximity to the tumor core during treatment exhibited greater activation, potentially accompanied by negative feedback upregulation of LAG3 expression. Furthermore, concurrent chemoradiotherapy (CCRT) appears to modify the local tumor microenvironment, possibly through cytokine-mediated mechanisms, leading to localized increases in LAG3 levels.

### Single-cell RNA sequencing reveals that LAG3 predicts immune activation in post-therapeutic tissues

**Fig. 7:**
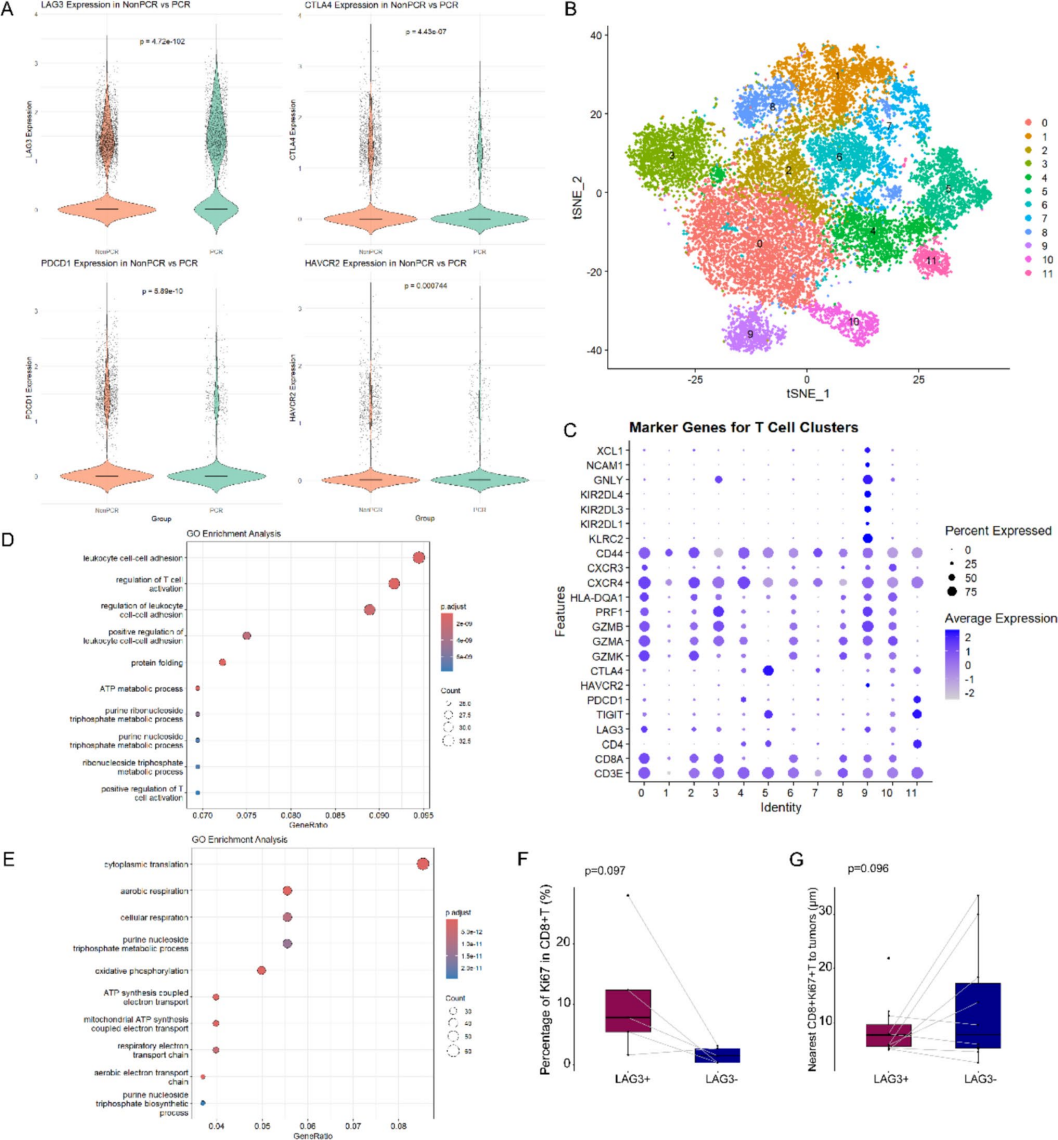
Immunofactor analysis of post-treatment tissues from surgical specimens. **A** ScRNA-seq violin plot showing differences in co-inhibitory receptor expression on CD8 + T cells in post-treatment tissues from the pCR and non-PCR groups. **B** T-SNE visualization and clustering of all single cells identified T cells. **C** Bubble plot of T cell subpopulations clustered based on marker genes. GOEA bubble map demonstrates LAG3 expression-associated pathways in **D** CD8 and **E** CD4 subpopulation T cells. **F** Ki67 expression rates in CD8 + T cells with LAG3 + vs. LAG3-. **G** Nearest distances from CD8 + Ki67 + T cells with LAG3 + vs. LAG3- to tumor cells. Spearman tests in (**A**, **F**, **G**), p < 0.05, statistically significant

We enrolled 10 patients who underwent neoadjuvant chemoradiotherapy (NCRT) followed by successful surgery at our hospital, and their surgical tissues were collected as post-treatment tissues. Two pathologists independently evaluated the tissues, identifying 3 cases as achieving pathologic complete response (pCR), and 7 cases with residual tumor cells as non-pathologic complete response (non-pCR). To investigate the differential expression of co-inhibitory receptors between the pCR and non-pCR groups, we performed single-cell RNA sequencing (scRNA-seq) on the post-treatment tissues. The results revealed that LAG3 expressions were significantly higher in the pCR group compared to the non-pCR group, whereas CTLA-4, PD-1, and HAVCR2 (TIM-3) exhibited elevated expression levels in the non-pCR group (p < 0.001, Fig. 7A). These findings suggest that LAG3 may function as an independent predictive factor associated with a favorable prognosis, distinct from other co-inhibitory receptors.

Subsequently, we performed a subpopulation analysis of T cells within the post-treatment tissues and broadly defined each subpopulation (Fig. 7B, C). We observed that among the T cell clusters defined by marker genes, the clusters expressing LAG3 were predominantly CD8-positive T cells. These cells exhibited a higher percentage of the key cytotoxic molecule perforin-1 (PRF1), indicating an activated anti-tumor immune response in this population. Based on the scRNA-seq results, the two subpopulations with high LAG3 expression both belonged to CD8 + T cells. One subpopulation expressed CD44 + CXCR3 + CXCR4 + PRF1 + and GZM family genes, while the other expressed XCL1 + NCAM1 + GNLY + and KIR family genes, suggesting that these T cells possess a robust capacity to mediate tumor cell apoptosis. It was noteworthy that in the tissue samples of this study, the CD4 + T cell subpopulation in post-treatment tissues exhibited lower expression of PRF1, suggesting immunosuppressive properties in these cells. Although these T cells did not show significant expression of the co-inhibitory receptor LAG3, they demonstrated higher levels of CTLA4, TIGIT, and PD1. This observation further implied that LAG3, as a co-inhibitory receptor, may play a more nuanced role in anti-tumor immune responses.

We next categorized T cells into two subpopulations, CD8-positive and CD4-positive, based on scRNA-seq results and performed gene ontology enrichment analysis (GOEA). The results showed that the expression of LAG3 in CD8 + T cells was associated with leukocyte cell–cell adhesion, T cell activation and protein folding (Fig. 7D). LAG3 expression in the CD4 subpopulation correlates with cytoplasmic translation, cellular respiration, and metabolic pathways such as oxidative phosphorylation, purine nucleoside triphosphate metabolism and biosynthesic processes (Fig. 7E). These findings suggested that LAG3 + T cells within the CD4 + subpopulation may exhibit stronger stemness and play a pivotal role in immune regulation.

Based on single-cell sequencing results, LAG3 expression was more sporadically distributed among T cells in post-treatment tissues. To further investigate the role of LAG3 in CD8 + T cells under non-stressful conditions following chemoradiotherapy, we conducted multiplex immunohistochemistry (mIHC) analysis of post-treatment tissues. The results demonstrated that CD8 + LAG3 + T cells exhibited a higher proliferative capacity compared to CD8 + T cells lacking LAG3 expression (11.06% vs 1.64%, p = 0.097, Fig. 7G). Additionally, within non-pCR tissues containing residual tumor cells, the distance between LAG3-expressing CD8 + Ki67 + T cells and tumor cells was significantly shorter than that of LAG3-negative T cells (6.77 μm vs 14.79 μm, p = 0.096, Fig. 7H). This suggests that LAG3-expressing CD8 + T cells may exert a more potent anti-tumor immune response.

## Discussion

The density of LAG3-positive cells and that of CD4 and CD8 positive cells showed a significant correlation in both baseline and on-treatment tissues, which corroborated the expression of the immune checkpoint LAG3 on activated T cells[3, 4]. Furthermore, the density of LAG3 + cells or the ratio of LAG3 positivity in a single subpopulation of active T cells was also associated with the expression of higher levels of Ki67, indicating a more active proliferation of effector cells. Based on this, we speculated that, unlike previous common findings, LAG3 showed a more active role in ESCC patients who underwent radical concurrent chemoradiotherapy. Notably, we observed that elevated LAG3 expression in treated ESCC tissues correlated with both prognostic improvement and indicators of immune activation—a surprising finding that contrasts with LAG3's classical'inhibitory'characterization. While extensive literature documents LAG3's immunosuppressive properties and negative clinical impact [36], emerging evidence, including in ESCC contexts, suggests its paradoxical association with immune activation and improved outcomes [15, 17, 37]. These findings do not negate LAG3's conventional inhibitory role but rather indicate that during CCRT in ESCC patients, microenvironmental cytokine changes may enable more sophisticated LAG3 regulation. Potential mechanisms include transient upregulation during intense T-cell activation, feedback mechanisms in activated T cells and context-dependent functionality where LAG3 may mark either activated or inhibitory states.

There is a clear correlation between higher expression of LAG3 in TIL and closer proximity of T cells to tumor cells, where LAG3 predicts more CD4 + or CD8 + T cells clustered around the tumor, and immune activation within the TME prompts more effector T cells to exert tumor-killing effects. Similarly, survival analyses showed that the presence of CD4 + or CD8 + T cell subsets in close proximity and clustering around tumor cells were both associated with a good prognosis in patients with ESCC, and whether these TILs expressed LAG3 in fact exerted some influence, suggesting that LAG3 exhibits a close association with immune activation in tissues treated with CCRT, even though LAG3-positive TILs in pre-treatment tissues seem to exhibit a trend towards closer resemblance to immunosuppression.

In our opinion, LAG3 seems feasible as a single efficacy predictor of CCRT in patients with ESCC, as demonstrated by survival analysis. The density of LAG3-positive cells in the TME is positively associated with better prognosis in both pre- and on-treatment tissues. Given that LAG3 is expressed in active CD4 + and CD8 + T cells, we can view its elevation as an outward manifestation of the presence of more effector lymphocytes in the tumor tissues, whereas more lymphocytes equate to more active tumor immunity and more benefits for ESCC patients[3, 12]. Consistency was also maintained in the relationship between the on/pretreatment ratios of CD4 + or CD8 + T cell density and LAG3 + cell density and survival. When we considered the expression rate of LAG3 in different subpopulations of TILs, LAG3 expression in CD8 + T cells showed a positive correlation with survival, whereas a significant negative correlation was observed in the pre-treatment CD4 + TIL population, which was might attributed to the fact that LAG3 exerts its distinctive function as an inhibitory immune checkpoint in CD4 + T cells[3, 21].

In the scRNA-seq results, LAG3 exhibited distinct roles in CD8 + and CD4 + T cell subpopulations. Perforin-1 (PRF1), a cytotoxic molecule known to enhance anti-tumor immune responses, showed a positive correlation with LAG3 expression in this study. Existing research indicates that PRF1 (perforin) expression correlates with multiple factors and plays a pivotal role in regulating tumor immune networks. Notably, its expression shows significant association with various immune exhaustion markers, including LAG3[26, 39]. Following CCRT-induced damage, tumor regression and subsequent remodeling of the immune microenvironment occur. The localized infiltration of immune cells may serve as a key mechanism for recruiting and sustaining PRF1 + CD8 + T cells to exert their anti-tumor effects. Furthermore, we identified two T cell subpopulations characterized by CD8 + LAG3 + expression. The identified subsets include granzyme K (GZMK) + CD8 + effector memory T (TEM) cells characterized by strong CD8 positivity and dominant expression of GZMK among granzyme family genes, and KIR + CD8 + NK-like T cells exhibiting relatively lower CD8 expression levels and elevated expression of killer immunoglobulin-like receptors (KIR family genes), killer cell lectin-like receptors (KLR genes), Granulysin (GNLY) and chemokine ligands (XCL1/XCL2)[27, 35], representing more active anti-tumor immune responses within the tissue. Regarding the immunological mechanisms of LAG3 in T cells, a study using a mouse model of type 1 diabetes demonstrated that, in the absence of the co-inhibitory receptor PD-1, LAG3 expression may promote the differentiation of islet CD8 + T cells from naïve to suppressive or even terminally differentiated states, limiting epitope spreading and interfering with antigen recognition to dampen immune responses [28]. For the role of LAG3 in CD4 + T cells, it has been reported that LAG3 in conventional CD4 + T cells (Tconvs) from the peripheral blood of patients with head and neck squamous cell carcinoma may restrict the local anti-tumor immune response of CD8 + T cells by inhibiting a disintegrin and metalloproteinase domain-containing protein 10 (ADAM10) [20]. Some studies suggest that suppressing CD4 + T cell proliferation is a key mechanism by which LAG3 exerts its immune regulatory function. LAG3 may spatially interact with the CD4 molecule and the T cell receptor (TCR)-CD3 complex, and its glutamic acid–proline-rich tandem repeat (‘EP’) motif may sequester lymphocyte-specific protein tyrosine kinase (Lck) bound to CD4, thereby inhibiting T cell development and immune responses [29]. However, there is still a lack of definitive research on the immune-activating role of LAG3 in the local tissues of ESCC patients. Studies have shown that human CD8 + T cells often express KIRs and NKG2 A in a mutually exclusive manner, and the absence of PD-1 and LAG3 allows NKG2 A to impede the anti-tumor immunity of CD8 + T cells [30, 31]. We speculate that LAG3 may mediate an as-yet-undefined pathway associated with the higher expression of anti-tumor KIRs in CD8 + T cells. Pre-treatment CD4 + T cells demonstrated limited cytotoxic activity, likely reflecting an enrichment of Th2 helper cells or regulatory T cells (Tregs) within this population. However, the observed increase in intratumoral IFN levels post-treatment suggests a potential phenotypic shift toward more active Th1-polarized CD4 + T cells. These activated Th1 cells may play an enhanced role in anti-tumor immunity through mechanistic details [38]. As for the immune-activating effects associated with LAG3 expression in CD4 + T cells in the treated tissues of this study, we propose that this reflects the presence of more surviving tumor-killing cells, such as activated effector T cells, in the local tumor microenvironment (TME) under CCRT-induced damage. The upregulation of LAG3 may be a feedback response to the presence of more activated T cells rather than the cause [3, 21, 22].

Immunosuppressive factors and the effect of CCRT on the tumor microenvironment in patients with ESCC were also investigated. IFN-γ, which is produced by a variety of cells including cytotoxic T and NK, is a multi-effector cytokine. In this experiment, we intended to determine the function of effector T cells in the tissues by IFN-γ expression[23]. Within baseline tissues, the density of each subpopulation of TILs in the tumor microenvironment showed a negative correlation with their IFN-γ expression ratios, and higher percentages of IFN-γ also decreased the number of effector T cells in the tumor periphery. IFN-γ played a role here as demonstrated in the study by Rowe and Payne, inducing the expression of PD-L1 on non-resting Ki67 +/Ki67-low cells, and the high expression of PD-L1 attenuated T-cell activation, constituting a factor of immunosuppression in ESCC tissues[23–25]. Rapidly proliferating cells, both cancer and active T cells expressing CD4 and CD8, were compromised by the CCRT treatment in our study. Though the percentage of Ki67 expression did not change with treatment, there was a significant increase in the ratio of IFN expression, suggesting that T cells, although damaged overall, showed no change in their proliferative capacity and retained their cytokine-secreting functions. The above-mentioned results indicated that CCRT had some remodeling effects on the TME, but overall, it did not completely inhibit effector T cells to perform their functions. Zhang et al. and Yan et al. showed that the number of CD4 + and CD8 + T cells did not significantly decrease after immunotherapy combined with concurrent chemoradiotherapy or radiotherapy alone[10, 13]. However, since neither study systematically investigated the expression of cytokines (particularly IFN), we cannot currently determine whether the observed IFN elevation results from negative feedback following local T-cell activation, or,

tumor microenvironmental changes in the absence of combined immunotherapy lead to IFN overexpression, subsequently inducing local immunosuppression. Further investigation in the ESCC context is required to establish the causal relationship underlying these immune responses. Based on these findings, we predict that effector T cells within the tumor microenvironment (TME) may be functionally better protected from the damage induced by CCRT following combination immunotherapy.

In recent years, anti-PD-1 combined with anti-LAG3 therapies have been tested and applied clinically for the treatment of malignant melanoma [11]. However, the impact of LAG3 on prognosis varies across different tumor types. Existing literature indicates that high LAG3 expression is associated with poorer prognosis in malignant melanoma and pancreatic cancer [17, 33, 34]. A study on breast cancer patients with liver or brain metastases who responded poorly to anti-PD-1 therapy found that targeting LAG3 in combination with other co-inhibitory receptors, such as TIGIT, represents a potential therapeutic strategy for tumors with low PD-1 expression [35]. In tumor regions where PD-1 is either blocked or PD-1 receptors are absent, Galectin-3 (encoded by LGALS3), produced by both tumor cells and macrophages, can interact with LAG3 on T cell surfaces to suppress IFN-γ secretion and induce T cell apoptosis [21]. Another animal study on tumor immunology found that while LAG3 blockade did not increase T cell infiltration, it doubled the proportion of tissue-localized TCRαβ + CD4-CD8-NK1.1- innate αβ T-cells (iαβTs)—these IL17-producing immune cells have been shown to possess significant anti-tumor effects [32, 35, 40]. These factors may collectively enhance localized T cell responses following LAG3 blockade therapy. Like breast cancer, ESCC is another tumor type where high LAG3 expression is either positively correlated with better prognosis or shows no clear association [15, 17]. This single-institution retrospective study has certain limitations that may affect the generalizability and accuracy of our findings include limited sample sizes for some experimental results and restricted analysis of functional markers, constraining our exploration of specific CD4 + and CD8 + T cell subsets and phenotypes. Given the unique characteristics of LAG3 observed in this study and the complex interplay of immune checkpoints in advanced cancers, our plan to incorporate additional functional markers (including MHC class II, PD-1/PD-L1, PRF1, Galectin-3, IL-4, IL-17, IL-21, IL-9, and IL-10) to characterize phenotypic changes in T cell subsets before/after ESCC treatment and determine their functional states (activated, helper, or inhibitory) in the future [38]. Conduct prospective multicenter studies investigating to combination anti-LAG3 immunotherapy for locally advanced ESCC and expanded multi-institutional cohorts and external datasets are required for validation of current findings, to learn more about specifically evaluate of LAG3's potential as a patient stratification biomarker and its therapeutic target specificity in ESCC immunotherapy.

## Conclusion

In conclusion, our findings suggest that the immune checkpoint LAG3 is closely associated with immune activation in the ESCC tumor microenvironment in terms of both overall content and spatial relationship and may serve as a potential predictor of overall survival and CCRT efficacy in ESCC patients. Concurrent chemoradiotherapy without combination immunotherapy resulted in some degree of suppression of the overall status of tumor-infiltrating T cells, and we expect that patients will benefit from rational combination immunotherapy.

## Supplementary Information

Below is the link to the electronic supplementary material.

Supplementary file1 (DOCX 560 KB)

## Data Availability

No datasets were generated or analysed during the current study.

## Abbreviations

ESCC: Esophageal squamous carcinoma
LAG3: Lymphocyte activation gene-3
CCRT: Concurrent chemoradiotherapy
mIHC: Multiplex immunohistochemical staining
PD-1: Programmed cell death protein 1
PD-L1: Programmed cell death ligand 1
IFN-γ: Interferon-gamma
TIL: Tumor infiltrating lymphocytes
TME: Tumor microenvironment
NR: Not reached
OS: Overall survival
pCR: Pathologic complete response
scRNA-seq: Single-cell RNA sequencing
GOEA: Gene ontology enrichment analysis
