# Supplementary material for: LAG3 as a marker of immune activation in esophageal squamous carcinoma treated with concurrent chemoradiotherapy
Source: Cancer Immunol Immunother. 2025 May 24;74(7):215. doi: 10.1007/s00262-025-04076-2 (PMC12103390; doi:10.1007/s00262-025-04076-2)
Supplement: Supplementary file 1 — Supplementary file1 (DOCX 560 KB) [file 262_2025_4076_MOESM1_ESM.docx]

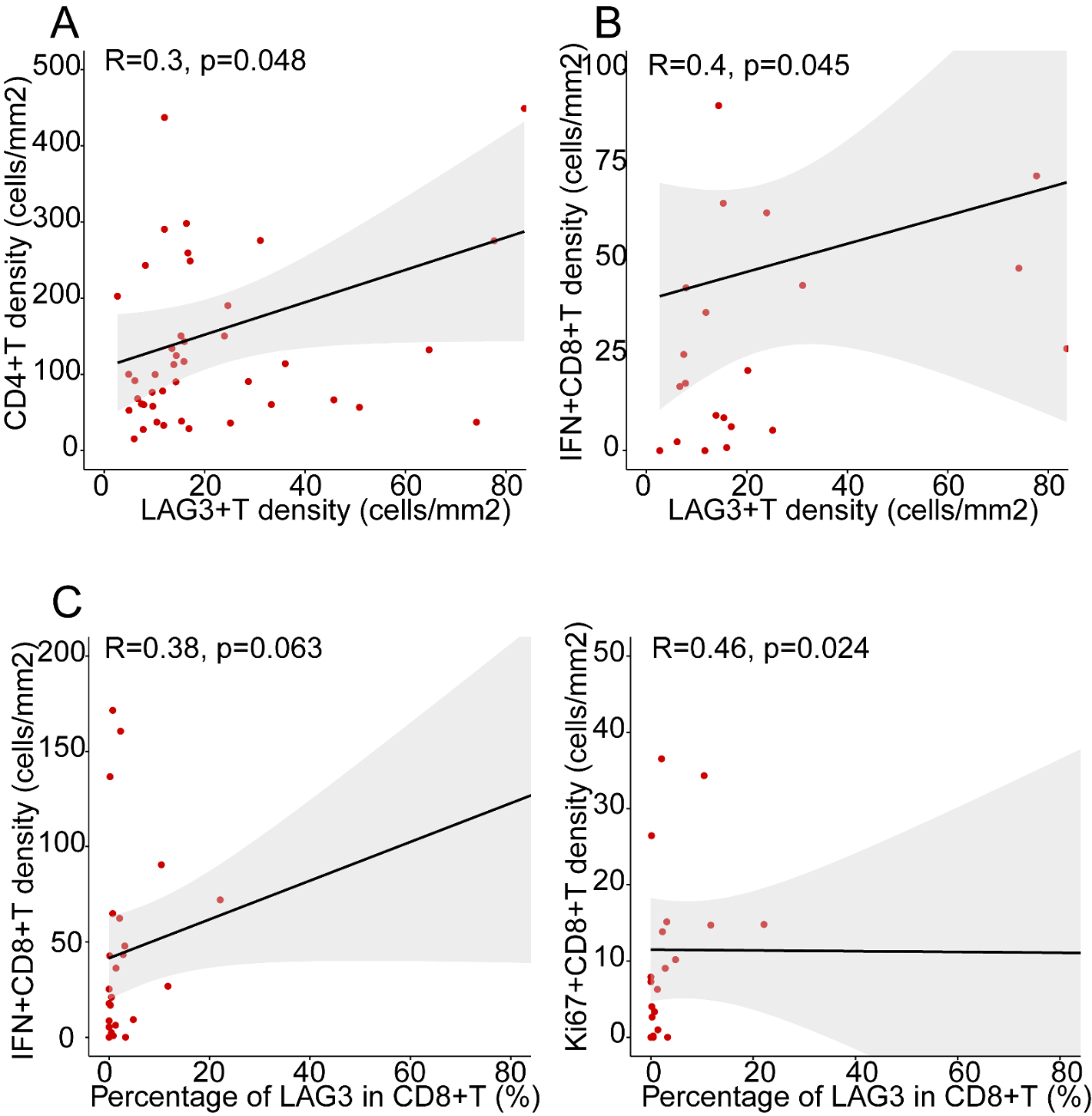


Figure Supplementary 1

T-cell distribution within the whole tissue at baseline. Relationship between (A) cell densities of CD4+ T cells, (B) IFN+CD8+ T cells and cell density of LAG3+ T cells. (C) Relationship between density of CD8+ T cells coexpressing IFN-γ or Ki67 and LAG3 expression rate in CD8+ cells. Spearman tests in (A-C), p<0.05, statistically significant.


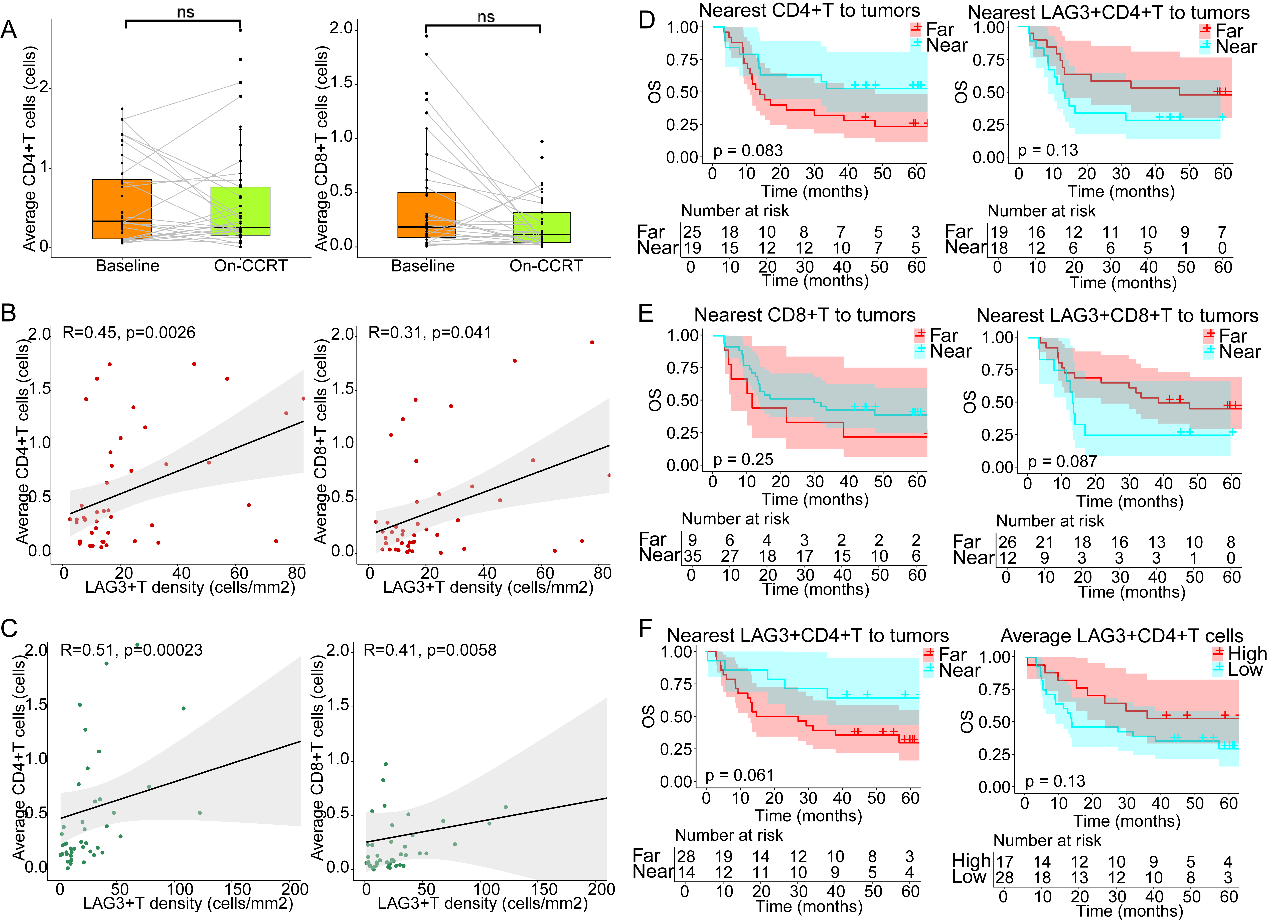


Figure Supplementary 2

Analysis of spatial distribution of T cells in the tumor microenvironment. (A) The average number of CD4+ and CD8+ T cells around tumor cells at baseline vs. on-treatment. Relationship between the average number of CD4+ and CD8+ T cells around tumor cells and density of LAG3+ T cells (B) at baseline and (C) on-treatment. Overall survival of patients based on (D) nearest distances from CD4+ and LAG3+CD4+ T cells to tumor cells and (E) nearest distances from CD8+ and LAG3+CD8+ T cells at baseline. (F) Overall survival on nearest distances from LAG3+CD4+ T cells to tumor cells and the average number of LAG3+CD4+ T cells around tumor cells on-CCRT. Wilcoxon signed-rank tests in (A). Spearman tests in (B-C). The individual TILs were divided into high content or far distances (redline) or low content or near distances (cyanline). Log-rank tests in (D-F). p<0.05, statistically significant.
